# Therapeutic Efficacy of Antibodies Lacking FcγR against Lethal Dengue Virus Infection Is Due to Neutralizing Potency and Blocking of Enhancing Antibodies

**DOI:** 10.1371/journal.ppat.1003157

**Published:** 2013-02-14

**Authors:** Katherine L. Williams, Soila Sukupolvi-Petty, Martina Beltramello, Syd Johnson, Federica Sallusto, Antonio Lanzavecchia, Michael S. Diamond, Eva Harris

**Affiliations:** 1 Division of Infectious Diseases and Vaccinology, School of Public Health, University of California, Berkeley, California, United States of America; 2 Departments of Medicine, Molecular Microbiology, Pathology & Immunology, Washington University School of Medicine, St. Louis, Missouri, United States of America; 3 Institute for Research in Biomedicine, Bellinzona, Switzerland; 4 Macrogenics, Inc., Rockville, Maryland, United States of America; Mount Sinai School of Medicine, United States of America

## Abstract

Dengue hemorrhagic fever and dengue shock syndrome (DHF/DSS) are life-threatening complications following infection with one of the four serotypes of dengue virus (DENV). At present, no vaccine or antiviral therapies are available against dengue. Here, we characterized a panel of eight human or mouse-human chimeric monoclonal antibodies (MAbs) and their modified variants lacking effector function and dissected the mechanism by which some protect against antibody-enhanced lethal DENV infection. We found that neutralizing modified MAbs that recognize the fusion loop or the A strand epitopes on domains II and III of the envelope protein, respectively, act therapeutically by competing with and/or displacing enhancing antibodies. By analyzing these relationships, we developed a novel *in vitro* suppression-of-enhancement assay that predicts the ability of modified MAbs to act therapeutically against antibody-enhanced disease *in vivo*. These studies provide new insight into the biology of DENV pathogenesis and the requirements for antibodies to treat lethal DENV disease.

## Author Summary

The four dengue virus serotypes (DENV1-4) cause the most prevalent mosquito-transmitted viral disease globally, infecting 50–100 million people annually in tropical and sub-tropical regions worldwide, yet no vaccine or therapy has been licensed to prevent or treat dengue. The greatest risk factor for severe dengue disease is a previous infection with a different serotype, which is thought to be due in part to a phenomenon known as antibody-dependent enhancement (ADE) whereby anti-DENV antibodies from a prior infection augment DENV infection of target Fcg receptor (FcgR)-expressing cells. We previously developed a mouse model that demonstrates antibody-enhanced lethal DENV disease and showed that genetically-modified antibodies incapable of interacting with the FcgR eliminate ADE *in vitro* and *in vivo*. In this study, we studied a larger panel of modified MAbs that recognize different regions of the DENV envelope protein. While all modified MAbs acted therapeutically to prevent a lethal, virus-only DENV infection, only certain MAbs effectively protected mice following an antibody-enhanced lethal infection. We determined that therapeutically effective MAbs following an ADE infection worked by competing for binding of enhancing antibodies on the DENV virion. Based on this, we designed an *in vitro* suppression-of-enhancement assay that predicted the ability of modified MAbs to act therapeutically *in vivo*.

## Introduction

The four serotypes of dengue virus (DENV) are transmitted by *Aedes aegypti* and *Ae. albopictus* mosquitoes and are endemic predominantly in tropical and sub-tropical regions of the world [1], [2]. Syndromes associated with DENV infection range from inapparent infection to classic dengue fever (DF), a debilitating self-limited disease, to life-threatening dengue hemorrhagic fever/dengue shock syndrome (DHF/DSS), characterized by vascular permeability and hypotensive shock [3]. Due to several factors, including geographic expansion of the DENV mosquito vectors and increased global urbanization, trade, and travel [4], [5], there has been a substantial increase in both the incidence of dengue epidemics and co-circulation of the four DENV serotypes in the same region [6]. This has resulted in an increased number of severe cases in dengue-endemic regions previously known for epidemics of only mild disease [1], [7]–[10]. While several tetravalent dengue vaccines are currently in various stages of clinical evaluation [11]–[14], no vaccine or therapy has been licensed to prevent or treat DENV-induced disease.

DENV is a member of the *Flavivirus* genus and is closely related to other medically important arboviruses including West Nile (WNV), Japanese encephalitis, tick-borne encephalitis, and yellow fever viruses [15], [16]. DENV has a 10.7-kb, positive-sense RNA genome with 5′ and 3′ untranslated regions flanking a polyprotein that encodes three structural and seven non-structural proteins [17]. Among the three structural proteins, the pre-membrane (prM/M) and envelope (E) proteins are the primary antigenic targets of the humoral immune response in humans [18]–[20]. The E protein is comprised of three domains (I (EDI), II (EDII) and III (EDIII) [21]–[24]), with EDII and EDIII containing the fusion peptide [25] and putative viral receptor binding site(s) [26], [27], respectively. For DENV, the most potently neutralizing antibodies generated in mice thus far target two sites on EDIII, corresponding to epitopes on the lateral ridge and A-strand [26], [28]–[31]. However, in human dengue-immune serum after primary DENV infection, highly neutralizing type-specific antibodies appear to be directed to quaternary epitopes on adjacent E proteins present only on virons [32]. A large proportion of human anti-DENV antibodies appear to be cross-reactive and to target the fusion loop or prM [18], [19].

Epidemiological analysis has established that a previous DENV infection is the greatest risk factor for the development of severe disease [33]–[37]. Infection with one serotype is believed to provide life-long immunity against re-infection with the same serotype but does not provide sustained protection against re-infection with a different serotype [38], [39]. Indeed, adaptive B and T cell responses may be poorly inhibitory against re-infection with a second serotype, and in a small percentage (∼1%) of cases, even exacerbate disease. One hypothesis, termed antibody-dependent enhancement, is that antibodies from a previous infection facilitate virus entry into Fcγ-receptor (FcγR)-bearing target cells, thereby increasing viral load and ultimately disease severity [40]. Experimental evidence in cell culture and in animal models supports this concept [41]–[44]. In a mouse model of ADE, passive transfer of monoclonal antibodies (MAb) or polyvalent serotype-cross-reactive serum, when administered at sub-neutralizing concentrations, was sufficient to enhance infection and cause lethal disease with DENV2 strain D2S10 in interferon α/β and γ-receptor deficient (AG129) mice [42], [43]. Recently, we showed that passive transfer of genetically engineered MAbs lacking binding to FcγR and C1q was sufficient to reduce viral load and TNF-α levels and to prevent lethal disease *in vivo*, even when administered one or two days after infection. Here, we evaluated the therapeutic activity of a larger panel of MAbs targeting different epitopes on the E protein following both a virus-only as well as an antibody-enhanced lethal infection. We determined that the two most potent therapeutic MAbs acted by competitively displacing either fusion-loop specific MAbs or enhancing polyclonal serum antibodies targeting a proximal epitope. Using this information, we designed a novel suppression-of-enhancement assay in human FcγRIIA-expressing K562 cells that predicts the ability of modified MAbs to act therapeutically against antibody-enhanced disease *in vivo*. Our observations provide new insight into the mechanism by which therapeutic MAbs prevent an antibody-enhanced lethal DENV infection.

## Results

### Neutralizing MAbs lacking effector functions prevent virus-only lethal disease

**Figure 1:**
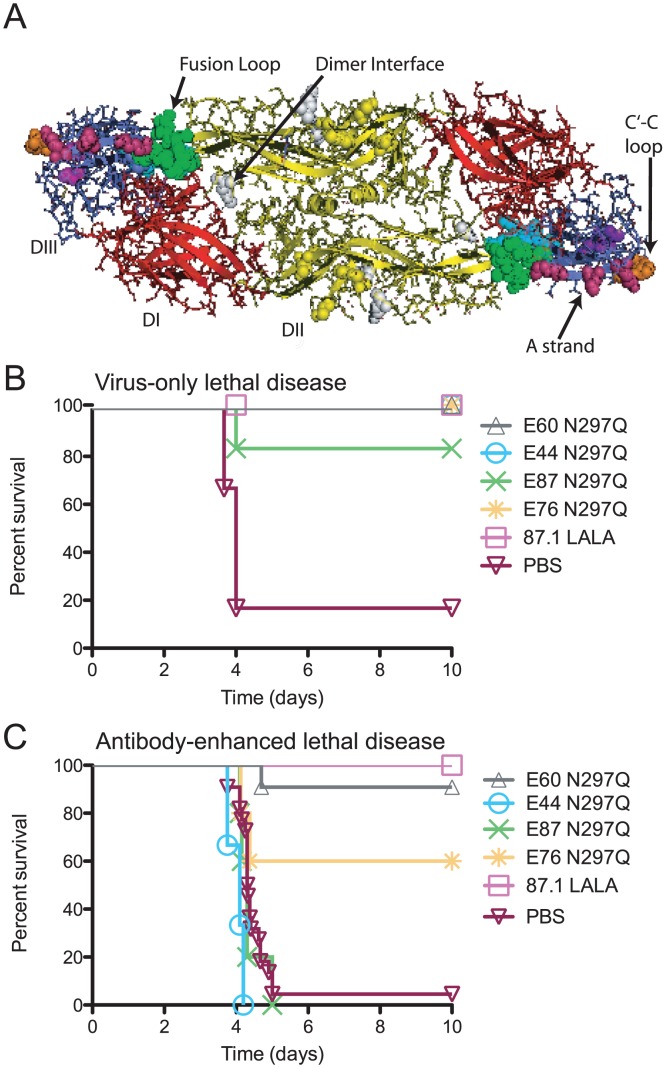
Anti-DENV MAbs are therapeutic following a virus-only or antibody-enhanced lethal infection. **A.** Ribbon diagram of the DENV2 E protein homodimer (PDB ID code 1OAN) [23]. EDI is red, EDII is yellow and EDIII is blue. The epitopes targeted by MAbs in Table 1 include the fusion loop (green), dimer interface (white), C-C′ loop (orange) and A strand (magenta). **B.** AG129 mice were administered a lethal dose of DENV2 D2S10 and 24 hours later were treated with 20 µg of modified MAbs (n = 5 per group from 2 independent experiments). **C.** AG129 mice were administered an enhancing dose of polyvalent DENV1-immune mouse serum, infected with DENV2 D2S10, and 24 hours later treated with 20 µg of modified MAbs. (n = 3–19 per group from at least 2 independent experiments for each modified MAb). See Table S1 for numbers of mice in each group. A Kaplan-Meier survival curve is shown (**B–C**), and log-rank analysis was used for statistical comparison.

Severe forms of DENV infection, including DHF/DSS, can be fatal, as no specific antiviral therapy is currently available. As such, we extended previous observations of the prophylactic and therapeutic efficacy of the EDII fusion loop-specific MAb E60 [42] by studying a larger panel of neutralizing MAbs targeting additional E protein epitopes, including the dimer interface (E44) on EDII and the C-C′ loop (E87) and A-strand (E76 and 87.1) on EDIII (**Figure 1A**).

Although secondary infection with a different DENV serotype is the greatest risk factor for severe DENV disease, DHF/DSS also has been reported following primary infection [45]. Thus, for a genetically-modified MAb to be a viable therapeutic option, it must protect following both a virus-only and an antibody-enhanced lethal DENV infection. To assess the ability of MAbs to protect in a direct model of lethal DENV infection, AG129 mice were infected with a lethal dose (4×10^6^ PFU) of DENV2 D2S10 and 24 hours later, administered 20 µg of individual genetically modified MAbs lacking the ability to bind FcγR or C1q. Notably, all of the modified MAbs tested prevented development of overt disease and protected against death in this model (*P*<0.05 for all MAbs as compared to untreated mice, **Figure 1B** **, Table S1**). We subsequently assessed whether these MAbs also protected against antibody-enhanced lethal DENV infection. Anti-DENV1 serum was administered 24 hours prior to a sub-lethal infection (10^5^ PFU) of DENV2 D2S10, and animals were treated 24 hours post-infection with 20 µg of genetically-modified MAbs lacking effector functions, where the N297Q variant MAbs are fully aglycosylated and the LALA variant MAbs remain glycosylated but incapable of binding either FcγR or C1q [42], [46]. Of those tested, only the EDII fusion loop-specific E60 N297Q and EDIII A strand-specific 87.1 LALA MAbs completely prevented mortality (*P*<0.01, **Figure 1C** **and Table S1**). In comparison, the EDIII-A-strand-specific MAb E76 N297Q showed partial protection (*P*<0.05), whereas MAbs E44 N297Q (EDII dimer interface) and E87 N297Q (EDIII C-C′ loop) provided no protection against lethal disease (**Figure 1C** **, Table S1**).

### Neutralization potency does not correlate with therapeutic efficacy across epitope classes *in vivo*

To determine why some MAbs had therapeutic activity in the virus-only lethal infection model but not in the context of antibody-enhanced infection, we examined several properties including epitope specificity, neutralization potency, and avidity. We first assessed whether neutralization potency correlated with *in vivo* therapeutic potential. The neutralizing activity against DENV2 D2S10 of each of the MAbs was assessed using a flow cytometry-based assay with human monocytic U937 cells expressing DC-SIGN, a known attachment factor for DENV [47]. The potency of each intact and modified MAb was assessed and expressed as the 50% neutralization titer (NT_50_ in ng/ml of MAb). No significant difference was observed between each intact MAb and its modified variant. The NT_50_ of therapeutically effective MAbs E60 N297Q (EDII fusion loop) and 87.1 LALA (EDIII A strand) were 72 ng/mL and 24 ng/mL, respectively. In comparison, the NT_50_ of MAbs E44 N297Q (EDIII C-C′ loop) and E87 N297Q (EDII dimer interface), which bound other epitopes in EDII and EDIII and lacked therapeutic activity, were similar (68 ng/mL and 95 ng/mL, respectively) (**Table 1**). Thus, NT_50_ values among MAbs targeting different epitopes failed to demonstrate a clear relationship between neutralizing potency and *in vivo* therapeutic efficacy in the context of antibody-enhanced lethal infections (Spearman ρ 0.47, *P* = 0.45).

**Table 1:** *In vitro* characteristics of intact and modified MAbs.

MAb	Source	Epitope	Cross-reactivity	Serotype	Average NT_50_ (ng/ml), non-modified^a^	Average NT_50_ (ng/ml), FcR-modified^a^	Kd_apparent_, non-modified MAb (nM)^b^	Kd_apparent_, FcR-modified MAb (nM)^b^	*In vivo* therapeutic outcome^c^: Lethal virus-only or ADE or Both
**E60**	27	EDII fusion loop	DV1,2,3,4	hIgG1	49	72	1.14	1.18	Both
**82.11**	19	EDII fusion loop	DV1,2,3,4	hIgG1	123	131	1.02	0.91	Virus-only
**E18**	27	EDII fusion loop	DV1,2,3,4	hIgG1	268	363	2.74	3.46	Virus-only
**E28**	27	EDII fusion loop	DV1,2,3,4	hIgG1	502	544	2.70	2.88	Virus-only
**87.1**	19	EDIII A strand	DV1,2,3	hIgG1	35	24	0.55	0.34	Both
**E76**	27	EDIII A strand	DV1,2	hIgG1	21	43	0.84	1.10	Both
**E44**	27	EDI/II dimer interface	DV2 only	hIgG1	122	68	0.14	0.84	Virus-only
**E87**	27	EDIII LR C-C′ loop	DV2 only	hIgG1	70	95	1.07	1.43	Virus-only
**E111**	27	Anti-DENV1	DV1 only	hIgG1	NN^d^	NN^d^	NB^e^	NB^e^	ND^g^
**22.3**	19	Anti-DENV4	DV4 only	hIgG1	NN^d^	NN^d^	ND^f^	ND^f^	ND^g^
**4G2**	ATCC	EDII fusion loop	DV1,2,3,4	mIgG2a	393	–	ND^f^	ND^f^	ND^g^

a The data presented is the average of two to five replicates of duplicate measures.

b The data presented is the average of two to four replicates of duplicate measures.

c “*In vivo* therapeutic outcome” refers to lethal, virus-only DENV2 D2S10 challenge or mouse anti-DENV1-enhanced DENV2 D2S10 infection (ADE).

d “NN” indicates that the MAb did not neutralize DENV2 D2S10.

e “NB” indicates that the MAb did not bind to DENV2 D2S10 by direct capture ELISA.

f “ND” indicates that the avidity for MAb 4G2 was not tested via direct capture ELISA.

g “ND” indicates that the therapeutic efficacy for these MAbs was not assessed under the conditions specified in footnote “c”.

### MAb avidity correlates with neutralization potency but not *in vivo* therapeutic efficacy across different epitopes

We hypothesized that MAb avidity, the strength of binding between a bivalent antibody and two ligands on a single virion or across virions, might correlate better with therapeutic efficacy following an antibody-enhanced lethal infection. To test this hypothesis, we measured the avidity (Kd_app_) of binding, using a direct, virion-coated ELISA [30]. While we noted a correlation between MAb neutralization titer and avidity (Spearman ρ 0.9, *P*<0.083), analogous to our neutralization data, we did not observe a relationship between MAb avidity and therapeutic efficacy (Spearman ρ 0.32, *P*<0.68) by MAbs targeting non-fusion loop epitopes (**Table 1**).

### Neutralization potency and avidity of MAbs that bind to the fusion loop epitope correlate with therapeutic activity following antibody-enhanced lethal disease

**Figure 2:**
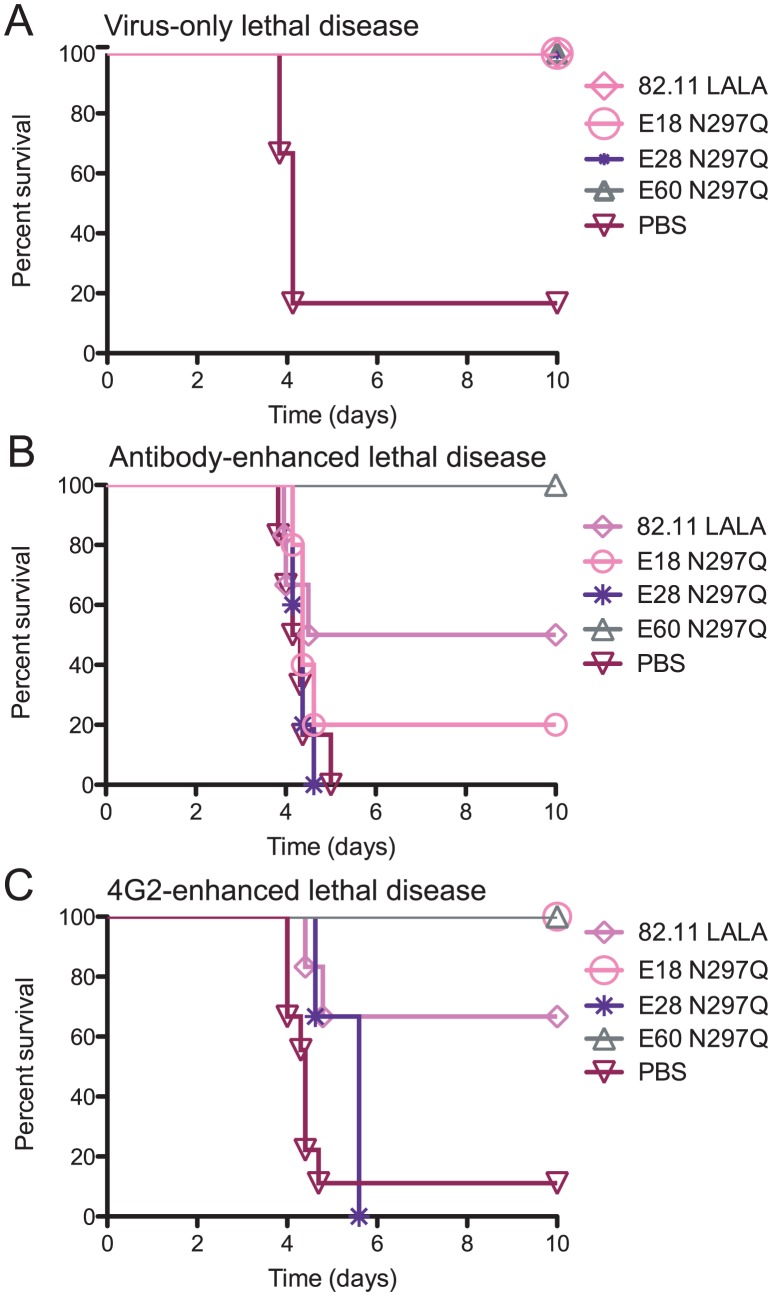
Modified MAbs targeting the fusion loop epitope prevent antibody-enhanced lethal DENV infection in relation to their neutralizing potency. **A.** AG129 mice were administered a lethal dose of DENV2 D2S10 and 24 hours later were treated with 20 µg of modified MAb targeting the fusion loop (n = 3–6 per group from 2 independent experiments). See Table S2 for numbers of mice in each group. **B.** AG129 mice were administered an enhancing dose of polyvalent DENV1-immune mouse serum, infected with DENV2 D2S10, and 24 hours later treated with 20 µg of modified MAbs (n = 3–6 per group from 2 independent experiments). See Table S2 for numbers of mice in each group. **C.** AG129 mice were administered an enhancing quantity (20 µg) of 4G2 MAb, infected with DENV2 D2S10, and 24 hours later treated with 20 µg of modified MAb (n = 3–9 per group from at least 2 independent experiments). See Table S3 for numbers of mice in each group. A Kaplan-Meier survival curve is shown, and log-rank analysis was used for statistical comparison.

As neutralization potency and avidity failed to correlate directly with the therapeutic efficacy of our modified MAbs across different epitopes, we investigated whether epitope specificity had greater predictive potential. As MAb E60 N297Q was highly protective even 48 hours following antibody-enhanced DENV infection [42], we hypothesized that the fusion loop epitope might be an important target for therapeutic MAbs. Therefore, we tested the therapeutic activity following either a virus-only or an antibody-enhanced lethal DENV infection of three additional modified MAbs that also target the EDII fusion loop but displayed between 2- to 8-fold reduced neutralization potency compared to MAb E60 N297Q: 82.11 LALA (NT_50_ 131 ng/ml), E18 N297Q (NT_50_ 363 ng/mL) and E28 N297Q (NT_50_ 544 ng/mL) (**Table 1**). Whereas all of the animals treated with MAb 82.11 LALA, E18 N297Q or E28 N297Q survived infection after a virus-only lethal challenge (*P*<0.01 compared to PBS-treated mice, **Figure 2A** and **Table S2**), MAbs E18 N297Q and E28 N297Q failed to confer a therapeutic benefit following an antibody-enhanced lethal infection (**Figure 2B** **, Table S2**). MAb 82.11 LALA protected 50% (3/6) of animals following an antibody-enhanced infection, though this difference trended but did not attain statistical significance compared to non-treated control animals (**Figure 2B** **, Table S2**). In contrast to the experiments with non-fusion loop-specific MAbs, studies with MAbs targeting the same fusion loop epitope suggest that neutralization potency can predict therapeutic efficacy following an antibody-enhanced infection (Spearman ρ 0.9487, *P*<0.083).

To explain the correlation between *in vitro* neutralizing potency and *in vivo* therapeutic efficacy within fusion loop-specific MAbs, we generated a model of competitive displacement. Recent work has suggested that a significant fraction of the human anti-flavivirus E protein antibody response is directed against the fusion loop epitope in EDII [20], [48]–[50]. We hypothesized that these cross-reactive antibodies found in DENV-immune serum are of intermediate or low affinity and bind to the heterologous virus at a stoichiometry insufficient for neutralization but adequate for enhancement of infection [51]. However, after administration of a therapeutic, high-affinity, genetically-modified fusion loop-specific MAb, natural dissociation of the enhancing antibody occurs, and the more avid therapeutic MAb binds to the fusion loop epitope, effectively preventing enhancing antibodies from binding again to the virion. Additionally, highly avid modified MAbs would compete favorably with enhancing antibodies for binding to nascently-produced virions. In this scenario, modified MAbs lacking effector functions either coat the virion allowing for direct neutralization or compete against cross-reactive fusion-loop enhancing antibodies in serum, such that the stoichiometry required for enhancement [51] is not reached. To test this hypothesis, we used 4G2, a weakly neutralizing (NT_50_ of 393 ng/mL) mouse MAb that binds to the fusion loop epitope [49] to enhance an otherwise sub-lethal DENV2 D2S10 infection and administered 20 µg of the modified MAbs 24 hours post-infection. E60 N297Q, the most therapeutic fusion loop-specific MAb in the context of a polyvalent serum-enhanced infection, again achieved 100% protection (*P*<0.01) when administered after a 4G2-enhanced infection, whereas MAb 82.11 LALA was less therapeutic (*P*<0.05), protecting 4 of 6 treated animals (**Figure 2C** **, Table S3**). None of the animals treated with MAb E18 N297Q succumbed to infection (*P*<0.05), although all demonstrated signs of illness (*P*<0.05 as compared to E60 N297Q-treated mice, **Table S3**). However, mice treated with MAb E28 N297Q all succumbed to 4G2-enhanced DENV2 D2S10 infection. These data support a model in which modified fusion loop-specific MAbs of sufficient avidity and neutralizing potency compete effectively for binding sites in the context of enhancing polyvalent DENV-immune serum or other fusion loop-specific MAbs to prevent disease.

### Modified fusion loop-specific MAbs compete with enhancing antibodies targeting the same or proximal epitopes

**Figure 3:**
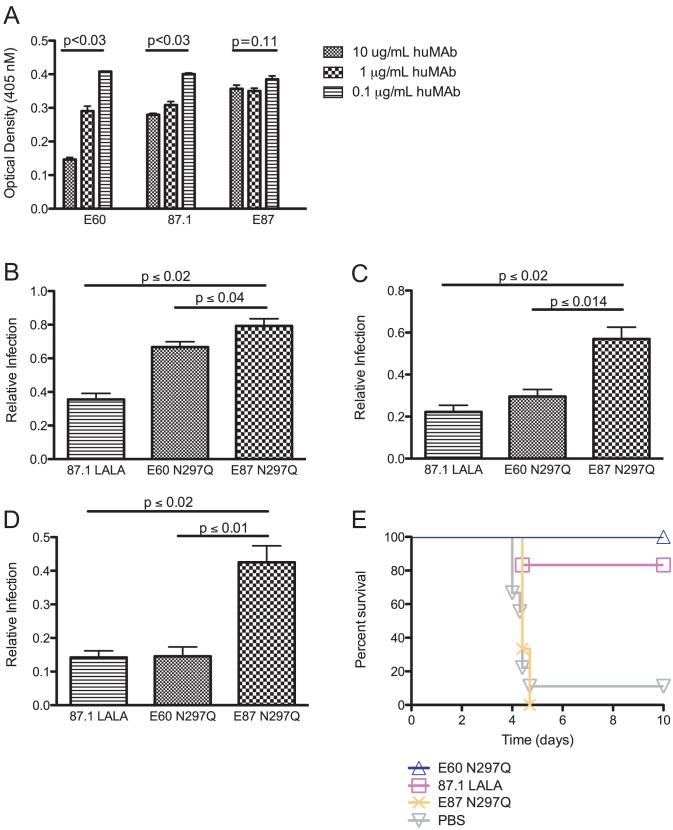
Fusion loop- and A strand-specific modified MAbs can compete for binding with fusion loop-specific MAbs of lesser neutralizing potency. **A.** Fusion loop-specific mouse MAb 4G2 was incubated at 1 µg/mL with MAbs E60, 87.1 or E87 at 10, 1 or 0.1 µg/mL human MAb prior to addition to DENV2-virion coated plates (for each MAb concentration, data is represented as mean +/− SEM). Anti-mouse, Fc-specific secondary MAb was then added, followed by PNPP substrate. Optical density (OD) values are shown on the y-axis and were calculated after subtracting the average background (binding of mouse Fcγ-chain specific secondary antibody in the absence of 4G2) from the raw OD. Statistically significant differences in 4G2 binding across the different human MAb concentrations were calculated using a Kruskal-Wallis analysis from triplicate values within each experiment. This data shown is representative of seven independent experiments. **B–D.** MAb 4G2 was pre-mixed with MAb E60 N297Q, MAb 87.1 LALA or MAb E87 N297Q in ratios of 95% 4G2/5% modified MAb (**B**), 85% 4G2/15% modified MAb (**C**), or 75% 4G2/25% modified MAb (**D**). For each 4G2/modified MAb mixture, a Gaussian distribution was used to fit the enhancement curve (**Figure S2**). The area under the curve (AUC) was calculated for each curve, and relative infection was expressed by dividing the AUC in the presence of modified MAbs by the AUC measured with 4G2 (no modified MAb) only. The data displayed are the average of three to seven independent experiments +/− SEM. Comparisons between the MAb combinations E87 N297Q/4G2 and E60 N297Q/4G2 or 87.1 LALA/4G2 were performed using a Kruskal-Wallis test. **E.** AG129 mice (n = 3–6 per group from one or two experiments) were administered an enhancing quantity (20 µg) of 4G2 MAb, infected with DENV2 D2S10, and 24 hours later treated with 20 µg of modified MAb. A Kaplan-Meier survival curve is shown, and log-rank analysis was used for statistical comparison.

We next evaluated directly whether MAb E60 (EDII fusion loop-specific) could effectively compete for binding with less potent fusion loop-specific MAbs, as compared to either therapeutic MAb 87.1 (EDIII A strand-specific) or non-therapeutic MAb E87 (EDII C-C′ loop-specific) that both target distinct epitopes. After directly coating DENV2 virions on microtiter plates, we added the moderately neutralizing mouse MAb 4G2 at 1 µg/mL mixed with increasing concentrations (0.1, 1 and 10 µg/mL) of modified human MAbs followed by an anti-mouse, Fc-specific secondary MAb. Binding of mouse MAb 4G2 was not affected by the amount of bound E87 (non-therapeutic, C-C′ loop-specific) (*P* = 0.64 by Friedman's analysis of data combined from seven experiments). In contrast, both MAb E60 (therapeutic, fusion loop-specific) and, surprisingly, MAb 87.1 (therapeutic, A strand-specific) altered binding of MAb 4G2; higher concentrations of MAb E60 and MAb 87.1 resulted in lower amounts of MAb 4G2 bound (*P*≤0.001 for both E60 and 87.1 by Friedman's analysis of data combined from seven experiments, **Figure 3A**). Less potently neutralizing and non-therapeutic fusion loop-specific MAbs competed as or less effectively against MAb 4G2 for binding to the fusion loop epitope (**Figure S1A**).

We next tested whether modified MAbs targeting the same or different epitopes with respect to the enhancing MAb 4G2 (fusion loop-specific MAb) could suppress enhancement *in vitro*. We mixed serial dilutions of both 4G2 and the modified MAbs E60 N297Q (therapeutic EDII fusion loop-specific, **Figure S2A**), 87.1 LALA (EDIII, A strand-specific, **Figure S2B**) and E87 N297Q (EDII dimer interface-specific, **Figure S2C**) in the following ratios: 100% 4G2; 95% 4G2 and 5% modified MAb; 85% 4G2 and 15% modified MAb; 75% 4G2 and 25% modified MAb. Each MAb combination was incubated with DENV2 D2S10 virus and used to infect K562 cells, a human erythroleukemic cell line that expresses FcγRIIA (CD32A) and is non-permissive in the absence of enhancing anti-DENV antibodies. Infection was monitored after 48 hours by intracellular DENV antigen staining and quantified by flow cytometry. Peak enhancement with MAb 4G2 occurred at 466 ng/mL and resulted in ∼7 to 15% of the cells becoming infected (**Figure S2**). However, when E60 N297Q or 87.1 LALA, MAbs that were effective therapeutically and lack the ability to engage Fcγ receptors, comprised only 5% of the MAb population, peak enhancement by 4G2 was reduced by 33% and 65%, respectively (**Figure 3B** **, Figure S2**). Remarkably, when 4G2 and the modified MAbs were mixed in a 75%: 25% ratio, E60 N297Q and 87.1 LALA both reduced infection by 85% and 86%, respectively (**Figure 3D** **, Figure S2**). In comparison, mixture of 5% of the non-therapeutic modified MAb E87 N297Q, reduced enhancement by only 21% (*P*<0.04, compared to E60 N297Q and *P*<0.02 compared to 87.1 LALA), and by 57% (*P*<0.01, compared to E60 N297Q and *P*<0.02 compared to 87.1 LALA) when a 75%: 25% mixture was used (**Figure 3B–3D**). Similarly to E87 N297Q, MAbs E18 N297Q and E28 N297Q, both fusion loop-specific but non-therapeutic MAbs, reduced enhancement by 19% and 6% when E18 N297Q and E28 N297Q were 5% of the MAb population, respectively, and by 46% when either E18 N297Q or E28 N297Q comprised 25% of the MAb population (**Figure S1B–D**). Thus, highly avid, fusion-loop specific MAb E60 N297Q and A-strand-specific MAb 87.1 LALA minimized *in vitro* enhancement, presumably by preventing binding of the enhancing fusion loop-specific MAb 4G2. Furthermore, the ability of the modified MAbs to prevent 4G2-mediated enhancement *in vitro* correlated with *in vivo* therapeutic activity in the context of anti-DENV polyvalent serum-enhanced infection.

Given the results in K562 cells with mixtures of modified MAbs and decreased 4G2-mediated enhancement, we wanted to evaluate further this relationship *in vivo*. We administered 20 µg of MAb 4G2 24 hours prior to infection with DENV2 D2S10 and then treated mice one day post-infection with 20 µg of MAb E60 N297Q (EDII fusion loop-specific)), 87.1 LALA (EDIII A strand-specific), or E87 N297Q (EDIII C-C′-loop-specific). While MAb E87 N297Q was not therapeutically protective against a 4G2-enhanced lethal infection, E60 N297Q and 87.1 LALA protected against mortality in 6 of 6 and 5 of 6 animals, respectively (*P*<0.05, **Figure 3E** **, Table S3**). Thus, potently neutralizing, fusion loop-specific (E60 N297Q) and A strand-specific (87.1 LALA) MAbs both prevent antibody-enhanced disease, likely by displacing binding of enhancing MAbs that target the fusion loop epitope.

### The stoichiometric relationship of modified and intact MAbs in relation to enhancement *in vitro* and *in vivo*

**Figure 4:**
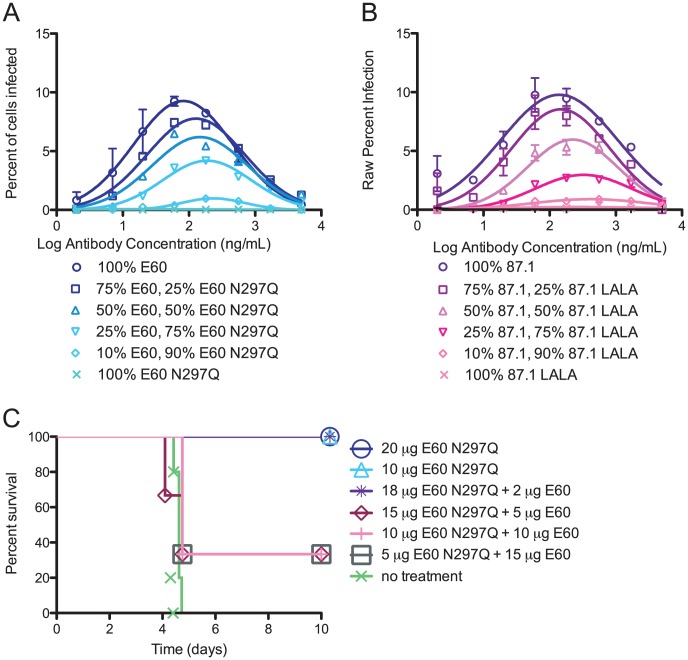
A high ratio of modified to non-modified MAb is necessary to prevent enhancement *in vitro* and *in vivo*. **A–B.** Non-modified and modified MAbs E60/E60 N297Q (**A**) and 87.1/87.1 LALA (**B**) were pre-mixed at ratios of 100% intact MAb, 75% intact:25% modified MAb, 50% intact:50% modified MAb, 25% intact:75% modified MAb and 10% intact:90% modified MAb. The data is plotted as the average of duplicate values where the absolute percent infection of K562 cells is shown on the y-axis. This data is representative of two or three independent experiments. **C.** AG129 mice (n = 3 per experimental group and n = 5 for non-treated control group) were administered a polyvalent DENV1-immune enhancing mouse serum, infected with DENV2 D2S10, and 24 hours later treated with a total of 20 µg of E60/E60 N297Q MAbs in the same combinations tested *in vitro* in (**A**). A Kaplan-Meier survival curve is shown, and log-rank analysis was used for statistical comparison.

We next assessed how different ratios of intact and genetically modified variants affected enhancement in K562 cells *in vitro*. We selected the two most therapeutically effective, modified MAbs (E60 N297Q (EDII fusion loop) and 87.1 LALA (EDIII A strand)), and mixed them with the intact parent MAbs in the following proportions: 100% intact MAb, 90% intact and 10% modified MAb, 75% intact and 25% modified MAb, 50% of each MAb, 25% intact and 75% modified MAb, 10% intact and 90% modified MAb, and 100% modified MAb. While several mixtures of E60 parent:E60 N297Q showed reduced enhancement, only the combination of 10% intact:90% modified was non-enhancing *in vitro*, suggesting that the majority of the antibody mixture must not bind to Fcγ receptors in order to abolish enhancement of DENV infection when MAb pairs are of comparable neutralizing potency and avidity (**Figure 4A**). The combination of intact 87.1 and 87.1 LALA also demonstrated a complete reduction in enhancement, but this occurred under conditions where a lower ratio of intact to modified mAb was required (ratios of 25% intact:75% aglycosylated (**Figure 4B**)). The relative differences in enhancement profiles observed between the E60:E60 N297Q and 87.1:87.1 LALA MAb pairs could be due to the small difference in the avidity and neutralization potency of the intact and modified MAb. Similar relationships between modified and intact MAb pairs were observed when studying MAbs that were moderately (E76/E76 N297Q) and poorly (E18/E18 N297Q) therapeutic (data not shown).

Using combinations of intact E60 and modified E60 N297Q (EDII fusion loop-specific MAb), we evaluated whether the requirement for 90% of the MAb mixture to lack FcγR binding for suppression of enhancement *in vitro* translated into therapeutic efficacy *in vivo*. The same ratios were mixed in a total of 20 µg and administered therapeutically 24 hours after serum-enhanced DENV2 infection of AG129 mice. Notably, and consistent with our data in K562 cells, complete therapeutic protection *in vivo* required 90% of the E60 mixture to be present in the modified form (*P*<0.02, **Figure 4C**). Mixtures that were combined in a ratio of less than 9∶1 showed reduced or no therapeutic efficacy (**Figure 4C**). This *in vivo* data suggests that when the same MAb is used for enhancement and therapy (intact versus modified), the majority of the mixture must lack the capacity for binding FcγR to avoid enhancement. Thus, a low stoichiometric threshold of binding is likely sufficient for enhancement of infection and disease.

### Suppression of enhancement in K562 cells *in vitro* predicts *in vivo* efficacy

**Figure 5:**
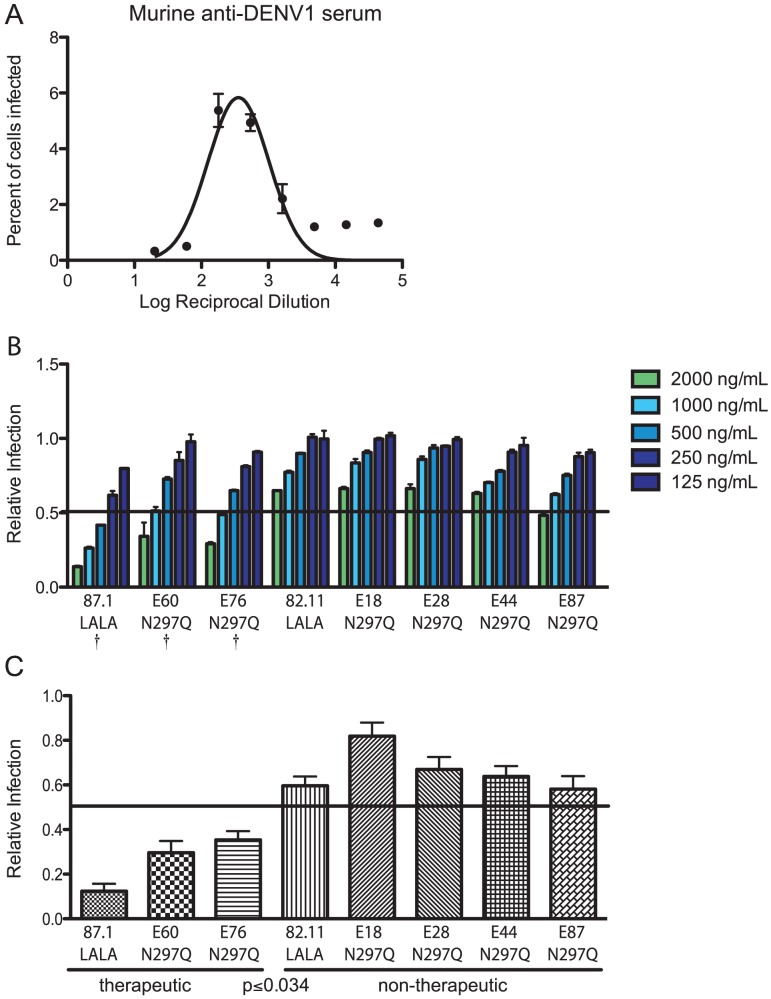
*In vitro* suppression-of-enhancement assay predicts therapeutic efficacy of MAbs *in vivo* with enhancing polyvalent DENV-immune serum from mice. **A.** The peak enhancing titer (PENT = 1∶180) for DENV1-immune mouse serum was determined in K562 cells. **B.** DENV1-immune mouse serum was diluted 1∶180 (PENT) and incubated with modified MAbs at six 2-fold dilutions beginning at 2,000 ng/mL. Relative infection was calculated by dividing the percent infection in the presence of modified MAbs by the percent infection measured with mouse DENV1-immune serum alone. The data displayed are the average of duplicate values and are representative of four independent experiments. A † indicates modified MAbs that are statistically therapeutic *in vivo* following mouse DENV1-enhanced, lethal DENV2 infection. **C.** The average infection across four experiments at 1,000 ng/mL of modified MAb (mean +/− SEM shown for each MAb). *P*<0.04 was obtained when comparing the average relative infection values for therapeutic to non-therapeutic MAbs using a Wilcoxon rank-sum analysis. The solid line indicates relative infection of 0.5 (50% infection).

Given the results with intact and modified MAbs, we evaluated whether we could use this *in vitro* relationship to predict the ability of modified MAbs to be therapeutically effective *in vivo* in the context of immune serum-enhanced DENV infection. Initially, using DENV1-immune mouse serum, we identified the serum dilution (1∶180) responsible for peak enhancement of DENV2 D2S10 in K562 cells (**Figure 5A**). We then tested the ability of modified MAbs to reduce enhancement in K562 cells by pre-incubating D2S10 with the peak enhancing dilution of DENV1-immune serum for 30 minutes, then adding increasing amounts of modified MAbs for 30 minutes, followed by incubation with K562 cells for 48 hours. Importantly, the concentrations of DENV-immune serum and virus used in the *in vitro* assay were comparable to those used in the *in vivo* infections. At concentrations of 2,000 ng/mL and 1,000 ng/mL, modified MAbs with moderate to strong (>60% protection) therapeutic activity *in vivo* were more efficient (*P*<0.05) at suppressing ADE in K562 cells than MAbs that were less therapeutically active (**Figure 5B and 5C**). The three most therapeutically effective MAbs (87.1 LALA, E60 N297Q and E76 N297Q) reduced enhancement on average by 88%, 70% and 65%, respectively, when added at 1,000 ng/mL while less protective MAbs (82.11 LALA, E44 N297Q, E87 N297Q, E18 N297Q and E28 N297Q) reduced enhancement less efficiently (**Figure 5C**). This trend also was observed when DENV1-immune serum was added at a different enhancing concentration (1∶540 dilution) (data not shown). Based on these data that differentiate *in vitro* therapeutic from non-therapeutic MAbs, we established ∼50% reduction at 1,000 ng/mL as the criterion for predicting therapeutic efficacy using the suppression-of-enhancement assay.

### Modified MAbs prevent enhancement by human DENV-immune serum in K562 cells and *in vivo*

**Figure 6:**
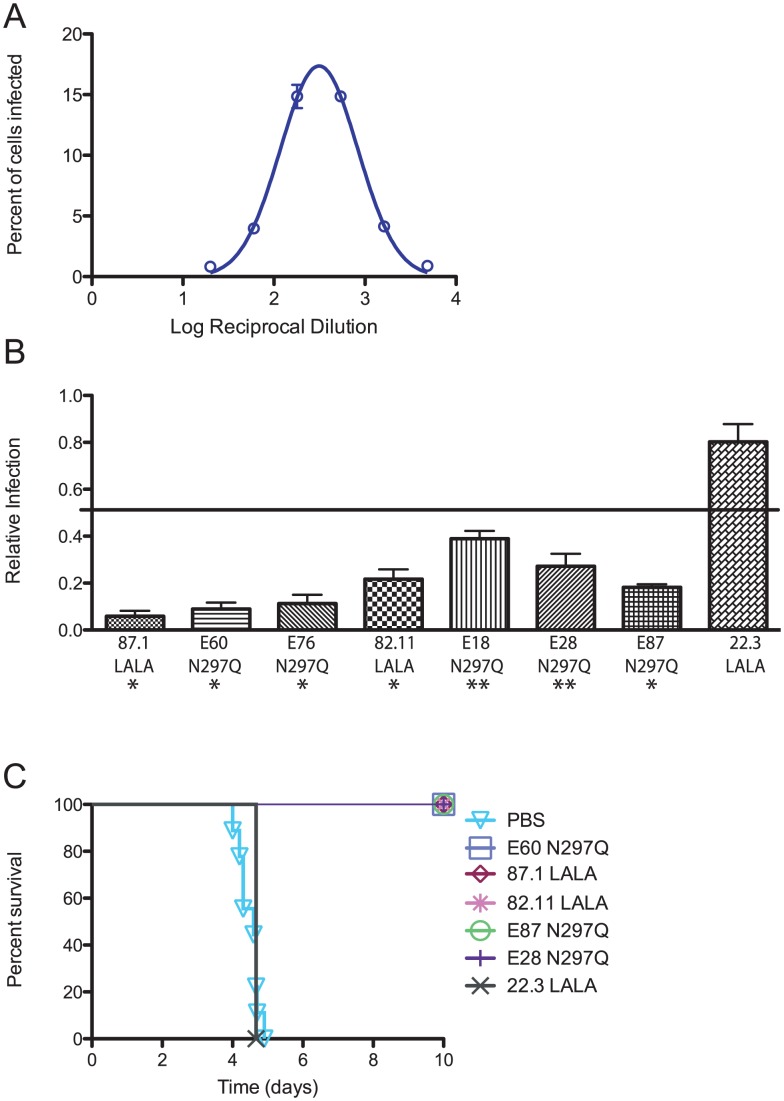
*In vitro* suppression-of-enhancement assay correlates with therapeutic efficacy of MAbs *in vivo* with enhancing polyvalent DENV-immune serum from humans. **A.** The PENT (1∶540) for DENV4-immune human serum was determined in K562 cells. **B.** DENV4-immune human serum diluted 1∶540 was incubated with modified MAbs at 1,000 ng/mL. Relative infection was calculated as described in **Figure 5**. The data displayed are combined from five independent experiments, and the mean +/− SEM is displayed for each MAb. A sign rank test was used to determine whether relative infection with each modified MAb was significantly lower than relative infection of 0.5 (50% infection), * *P*<0.05, ** *P*<0.08. **C.** AG129 mice (n = 3 per experimental group and n = 6 for non-treated control group) were administered an enhancing dose of DENV4-immune human serum, infected with DENV2 D2S10, and 24 hours later treated with 20 µg of modified MAbs. A Kaplan-Meier survival curve is shown, and log-rank analysis was used for statistical comparison.

As the K562 cell-based assay with mouse polyclonal anti-DENV1 serum and modified MAbs appeared to predict *in vivo* outcomes, we repeated the experiments with DENV-immune human serum; this was important as humans and mice produce overlapping yet distinct antibody repertoires against flavivirus epitopes [19], [48], [52], [53]. We evaluated whether modified MAbs reduced enhancement in K562 cells using human DENV-immune serum collected years after a primary DENV4 infection. The peak serum enhancement dilution again was identified as between 1∶180 and 1∶540 (**Figure 6A**). In contrast to the limited protection provided by the modified MAbs following a mouse DENV1-serum enhanced infection, most modified MAbs suppressed enhancement by DENV4 human immune serum below the 50% cut-off at the higher (*P*<0.05; E18 N297Q and E28 N297Q, *P*<0.08), yet physiologically relevant concentrations (1 and 2 µg/mL) of modified MAb (**Figure 6B** **, Figure S3**), while non-binding, DENV4-specific MAb 22.3 LALA did not (**Figure 6B**). Similar results were obtained when primary DENV1 or DENV3 human immune serum was tested (**Figure S3**).

To determine whether the enhancement data with human serum predicted protection *in vivo*, we administered normal human serum (NHS) or enhancing amounts of anti-DENV4 human immune serum 24 hours prior to a sub-lethal infection with DENV2 D2S10, and tested the therapeutic efficacy of the modified MAbs. As expected, all mice pre-treated with NHS survived infection without any signs of morbidity. All mice receiving enhancing anti-DENV4 human immune serum and treated with a modified MAb (E60 N297Q, 87.1 LALA, 82.11 LALA, E87 N297Q, and E28 N297Q) survived lethal enhanced infection with minimal signs of disease (*P*<0.05 for all modified MAbs compared to PBS-treated controls, **Figure 6C**), whereas mice treated with modified, DENV4-specific MAb 22.3 LALA did not (**Figure 6C**). Thus, the suppression-of-enhancement assay in K562 cells correlated with the therapeutic efficacy of modified MAbs *in vivo* in an antibody-enhanced lethal DENV model in the context of both mouse and human DENV immune serum. Moreover, and for reasons that likely relate to the distinct repertoire of cross-reactive enhancing antibodies in human serum, modified MAbs against the EDIII A-strand, EDIII C-C′ loop, and EDII fusion loop all efficiently suppressed antibody enhancement in cell culture and *in vivo*.

## Discussion

In this report, we analyzed a panel of eight MAbs that bind to several epitopes on the dengue virion, including the fusion loop and dimer interface on EDII and the A strand and C-C′ loop on EDIII. We determined that differences exist between the ability of modified MAbs lacking the capacity to engage FcγR and C1q to act therapeutically following a virus-only lethal infection and an antibody-enhanced lethal infection. Analysis of MAb characteristics such as binding avidity and neutralization potency did not clearly define an *in vitro* correlate of *in vivo* efficacy across different epitopes, but were more predictive when studying MAbs targeting a specific class, such as those binding the fusion loop epitope. Further analysis suggested that modified, fusion loop- and A-strand-specific MAbs act therapeutically by competing against enhancing antibodies in polyvalent serum that recognize the same or proximal epitopes. By studying these relationships, for the first time, we established a novel *in vitro* suppression-of-enhancement assay with polyclonal mouse and human anti-DENV immune serum that appears to predict the ability of modified MAbs to act therapeutically against ADE *in vivo*. Thus, we provide *in vivo* data that support *in vitro* observations about the mechanism of ADE as well as a means to suppress ADE *in vivo*.

Multiple parameters, including neutralization potency, avidity and epitope specificity, affect whether a modified MAb is therapeutic against an antibody-enhanced DENV infection. In our panel, in addition to binding to either the fusion loop or A-strand epitope, a therapeutic MAb needed to be strongly neutralizing (NT_50_<100 ng/mL), which itself is a function of epitope accessibility on the virion, mechanism of inhibition, and avidity of binding [51]. Four of the MAbs tested (E60, 82.11, E18, and E28) recognize similar residues within the EDII fusion loop ([54] and S. Sukupolvi-Petty and M.S. Diamond, unpublished data), but two (E18 and E28) had lower neutralizing potency and avidity of binding to the virion, and, correspondingly, showed less or no therapeutic activity *in vivo* following DENV enhancement by polyvalent mouse serum. While the avidity of binding to solid-phase DENV2 for 82.11 LALA and E60 N297Q was comparable, E60 N297Q is ∼2.5 fold more neutralizing, suggesting that the two MAbs might bind overlapping yet slightly distinct epitopes, or that the ensemble of viral conformations in solution [55] allows for enhanced recognition of E60 relative to 82.11. Analogously, when comparing two modified MAbs targeting the A-strand in EDIII, MAb 87.1 LALA showed higher avidity of binding and therapeutic efficacy *in vivo* compared to MAb E76 N297Q. Although further study is warranted, our data suggest that within an epitope class, there is a direct relationship between MAb avidity and neutralization potential *in vitro* and therapeutic efficacy *in vivo*.

Studies comparing therapeutic efficacy following virus-only and mouse antibody-enhanced lethal DENV2 infections revealed that all modified MAbs tested were therapeutic following a virus-only infection, but only two (E60 N297Q and 87.1 LALA) were completely protective following antibody-enhanced infection with DENV1-immune mouse serum. This observation suggests a direct interplay between the enhancing antibodies in polyvalent serum and the neutralizing therapeutic MAbs that determines outcome. Thus, a second parameter affecting therapeutic efficacy is the ability of a modified MAb to out-compete the enhancing antibodies in polyvalent immune serum for binding to the virion. This concept is supported by functional data *in vitro* and *in vivo* using the weakly neutralizing, fusion loop-specific MAb 4G2 and a panel of modified fusion loop-specific MAbs. In cellular assays, the more avid and strongly neutralizing MAb E60 N297Q was more effective at suppressing 4G2-enhanced infection in K562 cells than the less potent E18 N297Q and E28 N297Q MAbs. Consistent with this, E60 N297Q but not E28 N297Q prevented mortality as a therapeutic when MAb 4G2 was used to enhance a sub-lethal DENV2 D2S10 infection. Our data support a model in which therapeutic activity occurs when high-affinity, modified MAbs can bind to virions and neutralize infection by competing with and/or displacing enhancing antibodies for binding to similar epitopes.

An additional parameter that likely affects therapeutic efficacy of a MAb against antibody-enhanced DENV infection is its mechanism of action: whether the MAb binds prior to or following attachment of the virion to the target cell. However, the interpretation is not straightforward, as mechanism of neutralization of a given MAb may be affected by several variables: (a) stoichiometry and relative fractional occupancy at a given concentration [51]; (b) cell type and repertoire of attachment ligands or receptors [51], [54]; (c) virus particle maturation [56]; and (d) dynamic state of the virion [55]. From *in vitro* ADE experiments using K562 cells and non-modified MAbs, we can conclude that all MAbs (excluding E44 as it was not available for these studies) have the capacity to neutralize infection via a post-attachment mechanism at saturating concentrations (**Figure 4** and **Figure S2**). Following uptake via FcγR, antibody-enhanced DENV may still be neutralized by MAbs that block post-attachment – this phenomenon of trans-dominant neutralization of ADE by MAbs was described previously with the anti-WNV MAb E16 [57], a MAb that neutralizes WNV infection by blocking the structural changes required for viral fusion [58], [59]. Indeed, at saturating concentrations, all of the non-modified WT MAbs in our panel reduce K562 infection to background levels (**Figures 4A and B**, and **Figure S2**, right side of the curve), most likely by blocking fusion, a critical step required for release of the DENV genome into the cytosol following receptor-mediated endocytosis. To distinguish this post-attachment neutralization pattern from a MAb that blocks via a pre-attachment mechanism and cannot prevent infection in a K562 assay, we can compare these data to the effects of anti-fusion loop MAb E60 on WNV infection. MAb E60 cannot prevent WNV infection in K562 cells even at saturating concentrations (E. Mehlhop and M.S. Diamond, unpublished data). The inability of MAb E60 to neutralize WNV in K562 cells occurs because WNV virions are present in a mature state to a far greater degree than DENV and therefore have a lower stoichiometry of binding for the fusion loop epitope [56], [60]. For WNV, MAb E60 fails to achieve a stoichiometry sufficient to block fusion of virus that has entered via FcγR-dependent enhancement. In contrast, in the current study, all MAbs appear to act in a post-attachment mechanism in K562 cells at saturating concentrations. However, *in vivo*, it is unlikely that the modified MAbs circulate at saturating concentrations, given the large amount of DENV virus and antigen present. Thus, the relevant question becomes which MAbs can reduce enhancement (generated by either polyvalent DENV-immune sera or MAbs such as fusion loop-specific 4G2) most efficiently when the modified MAbs are at sub-saturating concentrations. Under these conditions, therapeutically effective MAbs (87.1 LALA and E60 N297Q) show greater efficacy than the other MAbs evaluated, likely due to their ability to compete for binding with enhancing antibodies, which interferes with FcγR crosslinking and limits DENV uptake and infection.

Remarkably, MAb 87.1 LALA, which maps to the A strand of EDIII, also was effective against anti-fusion loop MAb 4G2-mediated lethal DENV infection. While not as potent as MAb E60, MAb 87.1 appeared to compete with fusion loop-specific MAb 4G2 in the solid-phase DENV2 ELISA and also suppressed 4G2-induced enhancement in K562 cells. One possible explanation is that the A-strand epitope on EDIII is located next to the EDII fusion loop on adjacent DENV E proteins within a dimer [23], such that on the virion in solution, high avidity binding of 87.1 LALA prevents lower-avidity fusion loop-specific enhancing MAbs (e.g., 4G2) from binding. While all MAbs tested appear to block DENV in a post-attachment mechanism at saturating concentrations, MAb 87.1 may be more potent at blocking fusion at sub-saturating concentrations than MAb E60. This hypothesis may explain why 87.1 LALA is more efficient at reducing 4G2-enhanced DENV infection in K562 cells, but less efficient at competing with MAb 4G2 in a fixed-virion ELISA than MAb E60 N297Q. Another possible explanation is that binding of the A-strand MAb 87.1 LALA alters the conformation of the mature DENV virion [61], [62], enhancing exposure of the fusion loop epitope and increasing binding and neutralization. Even though E87 N297Q is unable to compete for binding with fusion loop-specific MAb 4G2 in the solid phase assay, it can still bind to the virion and contribute to the stoichiometry required to neutralize DENV, thus accounting for the ∼50% reduction in *in vitro* enhancement when E87 N297Q comprises 25% of the antibody mixture. Despite this, E87 N297Q did not have therapeutic activity *in vivo* when 4G2 was used as the enhancing MAb. In addition, MAb E87 N297Q was less efficient at reducing MAb 4G2-enhanced infection than the therapeutically effective MAbs E60 N297Q and 87.1 LALA at any of the three conditions tested (5%, 15% or 25% modified MAb). Although more study is warranted, we speculate that the MAbs which bind epitopes that do not displace 4G2 enhancing MAbs did not protect *in vivo* because they failed to reach a stoichiometry that was sufficient for neutralization or do not block a post-attachment step (e.g., viral fusion).

Previous studies have established that the epitope repertoire of anti-flavivirus neutralizing antibody in mouse and human serum is different. Mice were found to generate neutralizing antibody responses against epitopes in EDIII (∼30%) [24], [26], [28], [30], [63], [64] that can be serotype-specific [29], [30], [65] or cross-reactive [28], [29], [31], [66]. In comparison, DENV-immune human serum preferentially targets the fusion loop epitope in EDII [52], [53] as well as complex quaternary epitopes near the EDI-DII hinge that span adjacent E proteins within a dimer [32], with little EDIII-specific neutralizing antibody generated (10–15%) [48], [64], [67], [68]. While it has not been explicitly studied, it seems plausible that the epitope repertoire for enhancing antibodies against DENV in human and mouse serum also vary. In support of this, we observed differences in the ability of modified MAbs to prevent antibody-enhanced lethal DENV infection when DENV-immune mouse or human serum was used. Only E60 N297Q, 87.1 LALA and E76 N297Q were therapeutically effective against infection enhanced with anti-DENV1-immune mouse serum. In contrast, all modified DENV2-reactive MAbs were therapeutic following an infection enhanced with DENV4-immune human serum. These data likely imply one of two non-mutually exclusive hypotheses: (a) cross-reactive enhancing MAbs present in DENV-immune human serum are weakly avid, such that higher affinity modified MAbs can bind and/or displace the enhancing antibodies, resulting in therapeutic protection *in vivo*; (b) cross-reactive enhancing MAbs present in DENV-immune human serum bind distinct epitopes, which do not interfere with binding and neutralization by modified MAbs targeting the EDII fusion loop, EDII dimer interface, EDIII A strand, or EDIII C-C′ epitopes. In possible support of this, recent studies of the human antibody repertoire against DENV suggest that anti-prM antibodies are a major component of the cross-reactive response and promote enhancement *in vivo* [18], [20]. Future studies that test the therapeutic efficacy of modified E protein MAbs in the presence of enhancing concentrations of prM-specific MAbs will be important to perform.

One limitation of this study is the passive transfer model used to develop lethal DENV disease; we tested limited concentrations of enhancing polyvalent immune serum to distinguish between therapeutic and non-therapeutic modified MAbs. In the future, a more detailed dose-response study with different enhancing sera or MAbs will be needed to determine the range of efficacy of modified MAbs in mediating protection. In addition, while the passive transfer model of enhancement and protection may be relevant for infant DHF/DSS where potentially enhancing antibodies are received passively *in utero*, it remains uncertain if similar principles apply during natural secondary DENV infection.

In summary, our results suggest a model in which neutralization, avidity, and epitope specificity contribute to the therapeutic efficacy of modified MAbs. Despite the differences between mouse and human polyvalent antibody repertoires, the suppression-of-enhancement assay in K562 cells accurately predicted *in vivo* therapeutic efficacy in both situations. While further study is needed, this assay could be used to screen additional modified MAbs for potential use as DENV therapeutics. Overall, given these promising results, we suggest that further exploration of the utility of modified MAbs as therapy for DENV infections is warranted.

## Materials and Methods

### Ethics statement

This study was carried out in strict accordance with the recommendations in the Guide for the Care and Use of Laboratory Animals of the National Institutes of Health. The protocol was approved by the Institutional Animal Care and Use Committee at the University of California Berkeley (R252-1012B).

### Viruses and cell lines

All viruses were propagated in *Aedes albopictus* C6/36 cells (American Type Culture Collection) and titered by plaque assay on baby hamster kidney cells (BHK21, clone 15) [69]. DENV2 D2S10 was generated as previously described [70]. All *in vitro* neutralization and enhancement assays and sub-lethal *in vivo* infections were performed with non-concentrated virus. DENV2 D2S10 virus was concentrated by ultra-centrifugation for use in the virion ELISA and by centrifugation using 100,000 MWCO Amicon filters (Millipore) for lethal, virus-only *in vivo* infections. U937-DC-SIGN (gift from A. de Silva, University of North Carolina, Chapel Hill) and K562 cells were used for flow cytometry-based *in vitro* neutralization [71] and enhancement assays [64], respectively.

### Generation and purification of mouse anti-DENV non-modified and aglycosylated MAbs

Mouse MAbs E60, E18 and E28 were generated against WNV E protein, but are cross-reactive with DENV E protein [54]. Anti-DENV2 MAb E44, E76, and E87 were generated against DENV2 and described previously [28]. All mouse MAbs were purified by protein A affinity chromatography (Invitrogen, Carlsbad, CA) and have been mapped previously [29], [72]. The generation of a chimeric human-mouse E60 MAb with the human IgG1 constant region and the mouse V_H_ and V_L_ region was performed as described previously [72]. The generation of chimeric E18, E28, E44, E76, and E87 MAbs was performed similarly. Point mutations in the Fc region (N297Q) that abolish Fcγ receptor and C1q binding were introduced by QuikChange mutagenesis (Stratagene). All recombinant MAbs were produced after transfection of HEK-293T cells, harvesting of supernatant, and purification by protein A affinity chromatography. The accession numbers for the sequences of the V_H_-V_L_ regions of the recombinant MAbs are as follows: E18_VL KC254882 E18_VH KC254883 E28_VL KC254884 E28_VH KC254885 E44_VL KC254886 E44_VH KC254887 E60_VL KC254888 E60_VH KC254889 E76_VL KC254890 E76_VH KC254891 E87_VL KC254892 E87_VH KC254893.

### Generation and purification of human anti-DENV MAbs

MAbs 87.1 and 82.11 are fully human MAbs, and their generation has been described previously [20]. Production of the LALA variants was performed according to a previously published protocol [20]. Recombinant MAbs were produced in HEK-293T cells and purified by sequential protein A affinity chromatography and size-exclusion chromatography. The accession numbers for the sequences of the V_H_-V_L_ regions of the recombinant human MAbs are as follows: DV82VH KC294013, DV82VL KC294014, DV87VH KC294015, DV87VL KC294016.

### Clinical serum samples from dengue patients

The single DENV1- and DENV3-immune human sera samples used in the *in vitro* suppression-of-ADE assay were de-identified and pre-collected as part of the Nicaraguan Pediatric Dengue Hospital-based Study [73]. Both serum samples were collected three months post-symptom onset and were obtained from individuals with a primary DENV infection [74], [75]. The protocol for the study was reviewed and approved by the Institutional Review Boards (IRB) of the University of California (UC), Berkeley, and of the Nicaraguan Ministry of Health. Parents or legal guardians of all subjects provided written informed consent, and subjects 6 years of age and older provided assent.

The primary DENV4-immune human serum sample used for *in vitro* suppression-of-ADE and *in vivo* ADE experiments was a gift from Dr. Aravinda de Silva (University of North Carolina (UNC), Chapel Hill) and were received as de-identified and pre-collected samples, and as such were not considered part of human subjects research by the IRB at UC Berkeley. Convalescent DENV-immune sera were obtained at UNC Chapel Hill from volunteers who had experienced natural DENV infections during travel abroad. The protocol for recruiting and collecting blood samples from returned travelers was approved by the IRB of UNC Chapel Hill. Written informed consent was obtained from all subjects before collecting blood.

### AG129 mouse infections

All procedures were pre-approved and conducted according to UC Berkeley Animal Care and Use Committee guidelines. AG129 mice [76] were bred at UC Berkeley.

### Production of mouse anti-DENV serum

AG129 mice were infected intraperitoneally (i.p.) with 10^5^ PFU of DENV1 strain 448 (gift from S. Kliks). Six to eight weeks post-infection, mice were sacrificed, and whole blood was collected by terminal cardiac puncture. Serum was isolated from whole blood by centrifugation, heat inactivated, and stored at −80°C.

### *In vivo* therapeutic experiments

***DENV2 D2S10 enhanced disease:*** AG129 mice were administered either 25 µl mouse anti-DENV1-immune serum or 200 µl human anti-DENV-4 immune serum or 20 µg of MAb 4G2 (in 200 µl final volume) i.p. 24 hours prior to infection with an intravenous (i.v.) sub-lethal, 10^5^ PFU dose of DENV2 D2S10. ***DENV2 D2S10 virus-only lethal disease:*** AG129 mice were infected i.v. with 4×10^6^ PFU of DENV2 D2S10. ***Treatment model:*** Mice were administered 25 µl of DENV1-immune serum on Day −1, 10^5^ PFU of DENV2 D2S10 on Day 0, and 20 µg of modified MAbs in a final volume of 100 µl i.v. 24 hours following infection (Day +1). All animals were monitored carefully for morbidity and mortality for 10 days following infection.

### DENV neutralization assay

The neutralization titer of each parent and modified MAb was measured using the U937-DC-SIGN flow cytometry-based neutralization assay as described [71]. NT_50_ titers were calculated as described previously [42]. Each NT_50_ titer is the average of between 3 and 5 individual experiments, with the exception of E44 N297Q (1 experiment).

### DENV enhancement assay

***Polyvalent serum:*** Enhancement curves were generated as described in [64]. Briefly, eight, three-fold dilutions of DENV-immune serum beginning at 1∶10 were pre-mixed with DENV2 D2S10 prior to addition to K562 cells. A Gaussian distribution was used to fit each enhancement curve, where percent infection was recorded on the y-axis and log-reciprocal serum dilution on the x-axis. The area under the curve (AUC) for each enhancement infection was calculated using Prism software. ***MAb competition:*** Intact and modified MAbs were pre-mixed in different ratios at a starting concentration of 40 µg/mL. Eight 3-fold dilutions were incubated in either duplicate or triplicate with DENV2 D2S10 at an MOI of 0.1 in a 1∶1 ratio before being added to K562 cells for an enhancement of infection assay [64].

### *In vitro* suppression-of-enhancement assay

DENV-immune serum was diluted to the concentration that resulted in the greatest enhancement of DENV2 infection in K562 cells. DENV2 D2S10 virus at an MOI of 0.1 and serum were mixed together in equal volumes for 30 to 45 minutes at 37°C. Modified MAbs were prepared in five 2-fold dilutions beginning at 2,000 ng/mL, added to the polyvalent serum/virus mixture, and incubated for an additional 30–45 minutes prior to the addition of 5×10^4^ K562 cells. The cells were washed 2 hours following infection and fixed and stained for viral antigen. Relative infection was expressed as the average percent infection for each duplicate divided by the percent infection measured without modified antibody (positive control, between 7 and 15%).

### Direct capture virion and competition ELISA

***Avidity ELISA*** **:** DENV2 D2S10 virus was isolated by ultra-centrifugation at 53,000× g for 2 hours at 4°C and resuspended in cold PBS with 20% FBS. Concentrated virus was diluted to 5×10^4^ pfu in carbonate coating buffer, pH 9.6, and 50 µl was added to each well of a 96-well flat-bottomed plate as described previously [30]. The plate was coated overnight at 4°C and washed thoroughly with PBS with 0.1% Tween-20 (PBS-T) prior to blocking (5% milk w/v in PBS-T) for one hour. Both the non-modified and modified MAbs were diluted to 120 µg/mL in blocking buffer and titrated two-fold for a total of 12 serial dilutions. Each MAb dilution was added in duplicate to the coated plate for one hour. The plates were washed with PBS-T and incubated with an alkaline phosphatase (AP)-conjugated goat anti-human secondary antibody (Meridian) and AP substrate PNPP (Sigma) for one hour each, with additional PBS-T washes in between each step. The reaction was developed for 45 minutes, and the absorbance was read at 405 nm on a UV-plate reader (Bio-Tek) using KC Junior software. ***Competition ELISA:*** Ninety-six-well flat-bottomed plates were coated with DENV2 as described above. Mouse MAb 4G2 was diluted to 1 µg/mL and mixed with human mAb diluted to 10, 1 and 0.1 µg/mL in a separate 96-well plate, and 100 µl of the mixture was added. After one hour, the plates were washed and incubated with goat anti-mouse Fcγ-specific biotinylated secondary (Jackson) followed by Streptavidin-AP (Invitrogen) and the plates were developed with PNPP as described above.

### Statistical analyses

All graphs were produced using GraphPad Prism 5 software (La Jolla, CA). Statistical analysis was performed using Stata v10 (College Station, TX) and Prism 5 software. Comparison between NT_50_ titers of non-modified and modified MAb pairs was conducted using a Wilcoxon rank-sum analysis. Comparison of survival rates was conducted using a non-parametric log rank test. A Spearman rho (ρ) was calculated to assess correlations between modified MAb NT_50_ titer, avidity, and therapeutic efficacy (0–100% survival). A Kruskal-Wallis test was used to compare 4G2 binding across increasing concentrations of human MAb, and a Friedman's analysis (matched pairs Kruskal-Wallis) was conducted by combining all data for each MAb tested. A Wilcoxon rank-sum test was used to compare differences in the percent reduction of 4G2-enhanced D2S10 infection in K562 cells with different mixtures of modified MAb as well as in the enhancement-suppression assay using mouse DENV1-immune serum to compare relative infection between therapeutic MAbs and non-therapeutic MAbs. A sign rank test was used to determine whether 1,000 ng/mL of modified MAb could reduce an infection enhanced with DENV4-immune serum significantly lower than 50%.

## Supporting Information

Figure S1 **Neutralizing potency contributes to competitive binding of fusion-loop specific MAbs.** **A.** **Mouse** MAb 4G2 (fusion loop-specific) was incubated at 1 µg/mL with anti-fusion loop MAbs 82.11, E18 or E28 at 10, 1 or 0.1 µg/mL human MAb prior to addition to DENV2-virion coated plates (for each MAb concentration, data is represented as mean +/− SEM). Anti-mouse, Fc-specific secondary MAb was then added, followed by PNPP substrate. Optical density (OD) values are shown on the y-axis and were calculated after subtracting the average background (binding of mouse Fcγ-chain-specific secondary antibody). Statistically significant differences in 4G2 binding across the different human MAb concentrations were calculated using a Kruskal-Wallis test from triplicate values within each experiment. These data are representative of three independent experiments. **B–D.** MAb 4G2 was pre-mixed with MAb E40 N297Q, MAb E18 N297Q or MAb E28 N297Q in ratios of 95% 4G2/5% modified MAb (**B**), 85% 4G2/15% modified MAb (**C**), or 75% 4G2/25% modified MAb (**D**). For each 4G2/modified MAb mixture, a Gaussian distribution was used to fit each enhancement curve. The area under the curve (AUC) was calculated for each curve, and relative infection was expressed by dividing the AUC in the presence of modified MAbs by the AUC measured with 4G2 only (no modified MAb). The data displayed are the average of three to seven independent experiments +/− SEM, and comparison between the MAb combinations E60 N297Q/4G2 and E18 N297Q/4G2 or E28 N297Q/4G2 was performed using a Kruskal-Wallis test.
(TIF)

Figure S2 ***In vitro*** **ADE assay with fusion loop-specific MAb 4G2 and modified MAbs.** MAbs E60 N297Q (**A**), 87.1 LALA (**B**) and E87 N297Q (**C**) were mixed with MAb 4G2 at the concentrations specified in a starting dilution of 40 µg/mL. The percent infection of K562 cells is shown on the y-axis and log antibody concentration (ng/mL) on the x-axis. A Gaussian distribution was used to fit each curve separately.
(EPS)

Figure S3 **Modified MAbs reduce** ***in vitro*** **enhancement by human DENV-immune serum.** DENV1-immune (**A**), DENV3-immune (**B**), and DENV4-immune (**C**) human serum were diluted 1∶540 (peak enhancement identified in **Figure 6A**) and incubated with modified MAbs at six 2-fold dilutions beginning at 2,000 ng/mL. Relative infection was calculated by dividing the percentage infection in the presence of modified MAbs by the percent infection measured with human immune serum alone. The data displayed are representative of three (DENV1-immune serum, DENV3-immune serum) or five (DENV4-immune serum) independent experiments. The dashed line indicates relative infection of 0.5 (50% infection).
(EPS)

Table S1 **Therapeutic efficacy of modified MAb variants targeting different epitopes.**
(DOC)

Table S2 **Therapeutic efficacy of modified MAb variants targeting the fusion loop.**
(DOC)

Table S3 **Therapeutic efficacy of modified MAb variants following 4G2-enhanced, lethal DENV2 D2S10 infection.**
(DOC)
