# Correction: Therapeutic Efficacy of Antibodies Lacking Fc. Receptor Binding against Lethal Dengue Virus Infection Is Due to Neutralizing Potency and Blocking of Enhancing Antibodies

**DOI:** 10.1371/annotation/e08f911a-15ec-46d0-bede-83fdf3af1801

**Published:** 2013-03-27

**Authors:** Katherine L. Williams, Soila Sukupolvi-Petty, Martina Beltramello, Syd Johnson, Federica Sallusto, Antonio Lanzavecchia, Michael S. Diamond, Eva Harris

There is an error in the article title, which is missing the word "binding". The title should instead read:

"Therapeutic Efficacy of Antibodies Lacking Fcγ Receptor Binding against Lethal Dengue Virus Infection Is Due to Neutralizing Potency and Blocking of Enhancing Antibodies"

