# Supplementary figures and images for: Therapeutic Efficacy of Antibodies Lacking FcγR against Lethal Dengue Virus Infection Is Due to Neutralizing Potency and Blocking of Enhancing Antibodies
Source: PLoS Pathog. 2013 Feb 14;9(2):e1003157. doi: 10.1371/journal.ppat.1003157 (PMC3573116; doi:10.1371/journal.ppat.1003157)

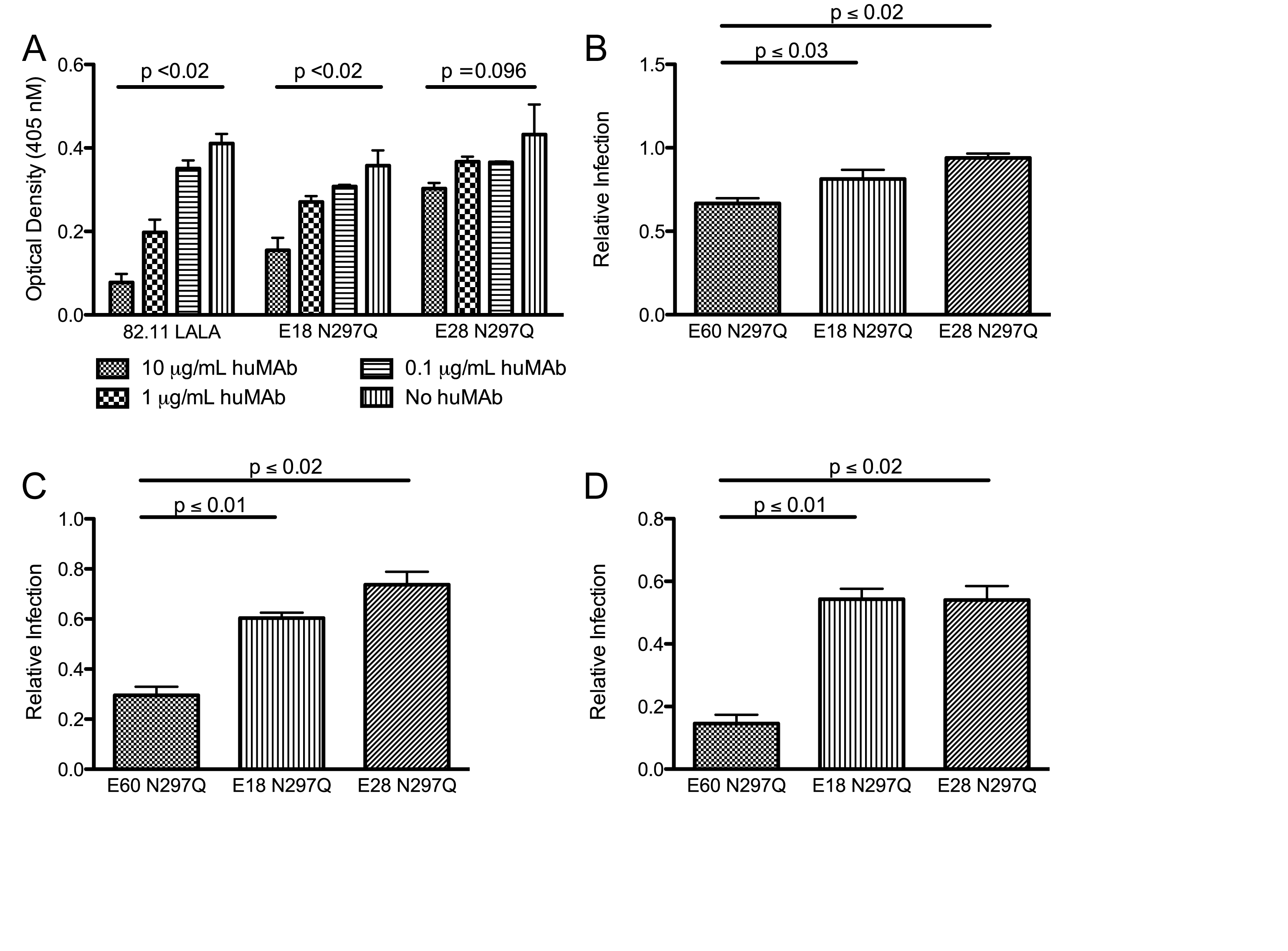

Supplement: Figure S1 — Neutralizing potency contributes to competitive binding of fusion-loop specific MAbs. A. Mouse MAb 4G2 (fusion loop-specific) was incubated at 1 µg/mL with anti-fusion loop MAbs 82.11, E18 or E28 at 10, 1 or 0.1 µg/mL human MAb prior to addition to DENV2-virion coated plates (for each MAb concentration, data is represented as mean +/− SEM). Anti-mouse, Fc-specific secondary MAb was then added, followed by PNPP substrate. Optical density (OD) values are shown on the y-axis and were calculated after subtracting the average background (binding of mouse Fcγ-chain-specific secondary antibody). Statistically significant differences in 4G2 binding across the different human MAb concentrations were calculated using a Kruskal-Wallis test from triplicate values within each experiment. These data are representative of three independent experiments. B–D. MAb 4G2 was pre-mixed with MAb E40 N297Q, MAb E18 N297Q or MAb E28 N297Q in ratios of 95% 4G2/5% modified MAb (B), 85% 4G2/15% modified MAb (C), or 75% 4G2/25% modified MAb (D). For each 4G2/modified MAb mixture, a Gaussian distribution was used to fit each enhancement curve. The area under the curve (AUC) was calculated for each curve, and relative infection was expressed by dividing the AUC in the presence of modified MAbs by the AUC measured with 4G2 only (no modified MAb). The data displayed are the average of three to seven independent experiments +/− SEM, and comparison between the MAb combinations E60 N297Q/4G2 and E18 N297Q/4G2 or E28 N297Q/4G2 was performed using a Kruskal-Wallis test. (TIF) [file ppat.1003157.s001.tif]
