# Supplementary material for: Therapeutic Efficacy of Antibodies Lacking FcγR against Lethal Dengue Virus Infection Is Due to Neutralizing Potency and Blocking of Enhancing Antibodies
Source: PLoS Pathog. 2013 Feb 14;9(2):e1003157. doi: 10.1371/journal.ppat.1003157 (PMC3573116; doi:10.1371/journal.ppat.1003157)
Supplement: Table S1 — Therapeutic efficacy of modified MAb variants targeting different epitopes. (DOC) [file ppat.1003157.s004.doc]

**Table S1. Therapeutic efficacy of modified MAb variants targeting different epitopes**

| **Modified MAb** | **Virus-only Mortality (n)** | **Virus-only Mortality p-value** | **ADE Mortality (n)** | **ADE Mortality p-value** |
| --- | --- | --- | --- | --- |
| 87.1 LALA | 0/6 | 0.0054 | 0/6 | 0.0002 |
| E44 N297Q | 0/3 | 0.037 | 3/3 | 0.94 |
| E60 N297Q | 0/6 | 0.0054 | 0/9 | 0.0001 |
| E76 N297Q | 0/8 | 0.0017 | 2/5 | 0.04 |
| E87 N297Q | 1/6 | 0.013 | 5/5 | 0.79 |
| PBS | 5/6 | --- | 18/19 | --- |

^a^ p-value vs. PBS-treated mice

^b^ p-value vs. PBS-treated mice
