# Supplementary material for: Therapeutic Efficacy of Antibodies Lacking FcγR against Lethal Dengue Virus Infection Is Due to Neutralizing Potency and Blocking of Enhancing Antibodies
Source: PLoS Pathog. 2013 Feb 14;9(2):e1003157. doi: 10.1371/journal.ppat.1003157 (PMC3573116; doi:10.1371/journal.ppat.1003157)
Supplement: Table S2 — Therapeutic efficacy of modified MAb variants targeting the fusion loop. (DOC) [file ppat.1003157.s005.doc]

**Table S2. Therapeutic efficacy of modified MAb variants targeting the fusion loop**

| **Modified MAb** | **Virus-only Mortality (n)** | **Virus-only Mortality** ^a^ **(p-value)** | **ADE Mortality (n)** | **ADE Mortality** ^b^ **(p-value)** |
| --- | --- | --- | --- | --- |
| E18 N297Q | 0/6 | 0.005 | 4/5 | 0.21 |
| E28 N297Q | 0/6 | 0.005 | 5/5 | 0.76 |
| 82.11 LALA | 0/5 |  | 3/6 | 0.19 |
| E60 N297Q | 0/3 | 0.04 | 0/3 | 0.01 |
| PBS | 5/6 | --- | 6/6 | --- |

^a^ p-value vs. PBS-treated mice

^b^ p-value vs. PBS-treated mice
