# Supplementary material for: Therapeutic Efficacy of Antibodies Lacking FcγR against Lethal Dengue Virus Infection Is Due to Neutralizing Potency and Blocking of Enhancing Antibodies
Source: PLoS Pathog. 2013 Feb 14;9(2):e1003157. doi: 10.1371/journal.ppat.1003157 (PMC3573116; doi:10.1371/journal.ppat.1003157)
Supplement: Table S3 — Therapeutic efficacy of modified MAb variants following 4G2-enhanced, lethal DENV2 D2S10 infection. (DOC) [file ppat.1003157.s006.doc]

**Table S3. Therapeutic efficacy of modified MAb variants following 4G2-enhanced, lethal DENV2 D2S10 infection**

| **Modified MAb** | **Morbidity (n)** | **Morbidity** ^a^  **(p-value)** | **Mortality (n)** | **Mortality** ^b^  **(p-value)** |
| --- | --- | --- | --- | --- |
| E18 N297Q | 3/3 | 0.65 | 0/3 | 0.02 |
| E28 N297Q | 3/3 | 0.19 | 3/3 | 0.07 |
| 82.11 LALA | 3/6 | 0.04 | 2/6 | 0.01 |
| 87.1 LALA | 2/6 | 0.03 | 1/6 | 0.007 |
| E44 N297Q | 2/3 | 0.59 | 2/3 | 0.20 |
| E87 N297Q | 3/3 | 0.2 | 3/3 | 0.60 |
| E60 N297Q | 0/6 | 0.0003 | 0/6 | 0.002 |
| PBS | 9/9 | --- | 8/9 | --- |

^a^ p-value vs. PBS-treated mice

^b^ p-value vs. PBS-treated mice
